# Regarding ‘Combination of reverse shock index and simplified motor score as a strong discriminator of trauma outcomes’

**DOI:** 10.1080/07853890.2025.2534092

**Published:** 2025-07-16

**Authors:** Bing Geng, Huazhen Bao

**Affiliations:** a Department of Neurology, Huantai County People’s Hospital, Qilu Hospital Huantai Branch, Huantai, China; b Department of Emergency Medicine, Yantai Penglai Traditional Chinese Medicine Hospital, Affiliated Hospital of Shandong College of Traditional Chinese Medicine, Yantai, China

To the editor

We read the article with great interest by Wu et al. [1], which presented a valuable study recommending the reverse shock index multiplied by the simplified motor score (rSI-sMS) for trauma outcome discrimination, with great interest. While the novel combination of haemodynamic and neurological markers is promising, we identified a critical methodological limitation affecting the interpretation of its purported superiority.

The authors exclusively used the area under the receiver operating characteristic curve (AUROC) to compare rSI-sMS with existing scores (e.g. rSI-GCS and rSI-GCSM). The AUROC can be used to evaluate discrimination but ignores calibration (agreement between predicted and observed risk). Table 3 shows that the rSI-sMS and rSI-GCS have nearly identical AUROCs for mortality (0.733 vs. 0.729, *p* = NS), ICU admission (0.605 vs. 0.608), and other endpoints. Crucially, no calibration metrics (e.g. Hosmer–Lemeshow test, calibration plots) were reported. This omission is especially relevant given the low event rates (e.g. 1.7% mortality rate), where models can exhibit good discrimination yet flawed risk estimation. A tool with an AUROC >0.7 may still stratify patients incorrectly if is poorly calibrated, leading to resource misallocation. Claims that the rSI-sMS outperforms the rSI-GCS lack statistical support when AUROC differences are negligible (*p* > 0.05) and calibration is unverified.

Calibration of the AUROC is critical because it directly affects the rational allocation of health care resources. Calibration reflects the consistency of the predicted probability with the actual probability of occurrence. When poorly calibrated, a model may systematically overestimate or underestimate risk (e.g. calibration slope deviation of 1.0 [2]), leading to biased clinical decision-making. For example, (1) The clinical overallocation of prophylactic resources (e.g. ICU beds and antimicrobial drugs) may result in wasted resources (calibration bias during periods of resource constraints [3] may exacerbate this problem) and thus the overestimation of their risks; (2) high-risk patients are not identified (e.g. undercalibration of RCRI scores for the prediction of cardiovascular events in ref. [4]), resulting in delays in or the lack of necessary interventions and consequently in the underestimation of their risks. Calibration optimization improves the efficiency of resource allocation by recalibrating a model and significantly boosting the net return [5].

Studies validating trauma scores emphasize calibration as a core metric. In two trauma scoring systems with similar discriminatory ability (AUROCs) (e.g. in this study, the AUROCs for mortality were 0.733 and 0.729 for the rSI-sMS and rSI-GCS, respectively), the calibration metrics were more important than the discriminatory ability [6]. When the discriminatory power is similar, calibration differences determine clinical applicability. The differences in the AUROC between the rSI-sMS and rSI-GCS in this study were not statistically significant (0.733 vs. 0.729, *p* > 0.05). Cook [7] demonstrated that the AUROC alone fails to capture model reliability in critical care settings. Similarly, the rSIG score [8] was used for both discrimination and calibration, ensuring robustness.

Future reanalysis should assess calibration using observed vs. expected outcome plots and the Hosmer–Lemeshow test and report the net reclassification improvement (NRI) or integrated calibration index (ICI) to quantify clinical utility beyond the AUROC. The following data points should be extracted from the trauma database: (1) Predictor variables: Raw continuous scores for each scoring system (e.g. the rSI-sMS, SI, mSI, rSI-GCS and rSI-GCSM values) used to generate predictive probabilities; (2) Outcome variables: dichotomous clinical outcomes (e.g. in-hospital mortality: yes/no; ICU admission: yes/no; ICU stay ≥14 days: yes/no; hospitalization ≥ 30 days: yes/no); and (3) Key covariates: Age (subgroups: < 65 years vs. ≥ 65 years); injury severity (ISS ≥16 vs. < 16); mechanism of injury (motor vehicle collision, fall); comorbidities (cardiovascular disease, no chronic disease); and TBI type (mixed vs. isolated brain injury).

Without calibration validation, the clinical applicability of the rSI-sMS remains unknown. Future studies must integrate calibration metrics to avoid overinterpreting differences in the AUROC and ensure safe implementation.

## Data Availability

Data sharing is not applicable to this article, as no new data were created or analysed in this study.
